# Efficacy and safety of haemopoietic stem cell transplant (HSCT) for mucopolysaccharidosis type I-Hurler syndrome (MPS-IH): a systematic review

**DOI:** 10.1186/s13287-026-05155-6

**Published:** 2026-08-12

**Authors:** Mubarak Patel, Karen Bean, Francis Pang, Miss Xiaoyu Tian, Miss Jiongyu Chen, Lisa Stirk, Nigel Armstrong

**Affiliations:** 1 Centre for Evidence and Implementation Science, Health Services Management Centre, University of Birmingham, Birmingham, UK; 2 Kleijnen Systematic Reviews Ltd, Escrick, York UK; 3 Orchard Therapeutics, London, UK; 4 Centre for Reviews and Dissemination, University of York, York, UK

**Keywords:** Mucopolysaccharidosis, Hurler, MPS-IH, Allogeneic, Haematopoietic stem cell transplant, HSCT, Long-term

## Abstract

**Background:**

Mucopolysaccharidosis type I-Hurler’s (MPS-IH) is the most severe form of MPS-I, with onset in infancy, followed by rapid neurodegeneration, and death typically within the first decade in untreated patients. A previous systematic review (SR) concluded that treatment with allogeneic haematopoietic stem-cell transplant (allo-HSCT) offers a clear survival and neurocognitive advantage, but does not prevent residual somatic disease. That review was published 15 years ago, which implies a need for a new SR to capture long-term efficacy, as well as safety outcomes reported since.

**Methods:**

We conducted a SR following guidelines of the Cochrane Collaboration and the Centre for Reviews and Dissemination. The search strategy had no date limits and was applied to multiple databases including MEDLINE and EMBASE. Searches were completed on 17.12.24. Reference checking ensured inclusion of all papers. All studies of allo-HSCT in MPS-IH were included if there were any efficacy outcomes with at least 5-years of follow-up, or any safety outcomes.

**Results:**

Thirty-five studies were included, none of which were comparative, 28 were retrospective and three included enzyme replacement therapy. Twenty-seven reported > 5-year efficacy, which showed survival was at least 80% in many studies (median follow-up between 3.8 and 9 years), including up to 62% at 10 years in one study, and 73.7% at 20 years in another. Neurocognitive and ambulatory preservation was observed in some patients, but in others there was progressive decline. Skeletal disease, poor ophthalmological outcomes with corneal opacity, cardiovascular disease, particularly affecting the heart valves, and pulmonary disease were also common. Nineteen studies reported safety, showing that graft versus host disease and pulmonary infections were common.

**Conclusion:**

Allo-HSCT appears to be an effective intervention for MPS-IH that can extend survival and preserve long-term neurological and physical function, particularly when administered early. However, although it remains the standard of care in modifying the disease course, skeletal, cardiovascular and sensory deficits often persist. The procedure also carries measurable risks, particularly related to mortality, GvHD and infection. Consequently, continued research and innovation are essential to improve long-term outcomes and quality of life for individuals with MPS‑IH.

**Supplementary Information:**

The online version contains supplementary material available at https://doi.org/10.1186/s13287-026-05155-6.

## Background

Mucopolysaccharidosis type I (MPS-I) is an autosomal recessive lysosomal storage disorder caused by deficiency of the enzyme α-L-iduronidase, resulting from biallelic pathogenic variants in the *IDUA* gene [1]. Enzyme deficiency leads to progressive accumulation of glycosaminoglycans (GAGs), predominantly heparan sulfate and dermatan sulfate, in multiple tissues [2]. This accumulation causes multisystem disease characterised by coarse facial features, hepatosplenomegaly, musculoskeletal abnormalities, valvular heart disease, corneal clouding, and progressive neurocognitive decline [3]. MPS-I has traditionally been divided into three clinical sub-types: the most severe phenotype, Hurler (MPS-IH), and the attenuated phenotypes, Hurler-Scheie (MPS-IH/S) and Scheie (MPS-IS) [4]. MPS-IH is the most severe form, with onset in infancy, followed by rapid neurodegeneration, and death typically within the first decade in untreated patients [5, 6].

Epidemiological studies confirm that although rare, MPS-IH represents one of the more frequently reported mucopolysaccharidoses. In the USA the incidence of MPS was found to be 0.98 per 100,000 live births, with a prevalence of 2.67 per 1 million [7]. A global incidence rate of MPS-IH has been reported to range from 0.69 to 1.66 per 100,000 births [8]. In England and Wales, Moore et al. [9] reported a birth prevalence of 1.07 per 100,000 live births for MPS-I overall, with 0.76 per 100,000 specifically for MPS-IH. Survival analysis from this cohort demonstrated a median survival of 8.7 years for MPS-IH patients, falling to 6.8 years when excluding patients who received allo-HSCT, reflecting the natural course of the disease in the absence of definitive treatment. More recent epidemiological literature of MPS-IH is limited since allogeneic haematopoietic stem cell transplantation (allo-HSCT) has been the standard of care for many decades.

The therapeutic rationale of allo-HSCT rests on cross-correction, whereby donor-derived cells engraft into tissues and secrete the enzyme α-L-iduronidase (IDUA), which can be taken up by deficient host cells. Donor stem cells can cross the blood brain barrier and differentiate into microglia-like cells, providing enzyme delivery across the blood–brain barrier [10]. This unique mechanism distinguishes allo-HSCT from intravenous enzyme replacement therapy (ERT), which does not have the same level of disease modifying effects as allo-HSCT [11]. Allo-HSCT is widely recognised as the current standard of care for MPS-IH. This position is supported by both the American Society for Transplantation and Cellular Therapy (ASTCT) [12] and the European Society for Blood and Marrow Transplantation (EBMT) [13], as early treatment with allo-HSCT has been shown to prolong survival, slow neurocognitive decline, and ameliorate several somatic manifestations. However, despite the use of optimised transplantation protocols, substantial limitations remain due to the allogeneic nature of the procedure. Treatment-related mortality has been reported between 5 and 20% [14–16], and post-transplant complications such as acute and chronic graft-versus-host disease [17, 18] carry significant morbidity, often requiring prolonged or lifelong immunosuppression. Moreover, numerous long-term follow-up studies have demonstrated a persistent and progressive burden of disease despite transplantation, including cognitive, neurological, ophthalmic, orthopaedic, cardiac, and respiratory complications [6, 19–21]. These residual manifestations substantially impact everyday functioning and quality of life for patients and their caregivers [21]. Thus, while allo-HSCT remains the cornerstone of disease-modifying treatment for MPS-IH, its limitations highlight the ongoing need for improved therapeutic strategies and earlier diagnosis.

A previous systematic review by van der Linden et al. [22] synthesised outcomes of allo-HSCT for MPS-IH and concluded that transplantation offers a clear survival and neurocognitive advantage (slowing/stabilisation of neurocognitive outcomes), particularly when performed at an early age, but does not prevent residual somatic disease. That review also emphasised the methodological limitations of the available evidence, including small cohort sizes, variable outcome measures, and lack of long-term follow-up. Since its publication, additional large cohort studies [23, 24] have provided further insights into long-term outcomes, safety, and evolving transplant strategies. In parallel, experimental approaches such as autologous gene-modified HSCT have shown encouraging preliminary results [25–27], underscoring the need to re-evaluate the current role of conventional allo-HSCT within the treatment landscape [25, 26].

Therefore, the aim of this systematic review was to provide an updated and comprehensive synthesis of the long-term efficacy and safety of allo-HSCT in MPS-IH.

## Methods

This review was conducted following guidelines recommended by the Cochrane Collaboration and the Centre for Reviews and Dissemination (CRD; York, United Kingdom [UK]), in order to reduce the risk of bias and error [28, 29]. The protocol has been registered as CRD42025648915 on the PROSPERO register (https://www.crd.york.ac.uk/PROSPERO).

### Search strategy and eligibility criteria

The search strategy was developed following consultation with topic and methodological experts. The search strategy did not have date limits, ensuring a thorough search of the literature was performed. The search strategy is presented in Additional file 1, and was applied to MEDLINE, EMBASE, the Cochrane Database of Systematic Reviews, the Cochrane Central Register of Controlled Trials and Northern Light Life Sciences Conference Abstracts. Completed and ongoing clinical trials were searched for using ClinicalTrials.gov, World Health Organization International Clinical Trials Registry Platform and Orphanet clinical trials. Reference checking ensured that all relevant papers had been retrieved. All database searches were completed on 17.12.24.

Studies were eligible for inclusion if they evaluated allo-HSCT in MPS-IH patients. Eligibility criteria were the following. The population of interest was individuals with a confirmed diagnosis of MPS-IH, and the intervention of interest was allo-HSCT, irrespective of donor source or conditioning regimen. To ensure adequate assessment of long term benefits, efficacy outcomes were restricted to studies that reported a minimum of 5 years post-transplant follow-up, and outcomes of interest included overall survival, pulmonary or cardiovascular function, hearing, vision, psychomotor or cognitive development, skeletal manifestations such as growth and facial features, activities of daily living, including school performance, and quality of life. Safety outcomes reported at any timepoint included adverse events (AEs), serious adverse events (SAEs), fatal AEs, and treatment-related complications such as viral infections, pulmonary haemorrhage, graft-versus-host disease (GvHD), or toxicities related to immunosuppressive or cytotoxic therapy. Eligible study designs were randomised controlled trials, prospective comparative studies, prospective single-arm studies, and prospective cohort studies. Retrospective analyses were included due to a large proportion of the literature > 5 years post allo-HSCT being based on, these study types. Case reports, narrative reviews, conference abstracts without any extractable data, and animal studies were excluded from this review.

### Study selection

Two reviewers independently screened all titles and abstracts of retrieved articles from the electronic database searches. Reviewers then repeated the process whilst screening the full texts of remaining papers. Any discrepancies were resolved via discussion, or by a third reviewer if conflict persisted. The data management process was performed using EndNote v.20 (Clarivate Analytics, USA).

### Data extraction

Data were extracted on population characteristics (age, sex and ethnicity), study characteristics (study design, study duration and sample size), efficacy outcomes (enzyme level, activities of daily living, cognitive function, psychometer development, skeletal abnormality, sensory function for both hearing and vision, quality of life (QOL), cardiovascular and pulmonary function, overall survival and event-free survival, etc.), and safety outcomes. To avoid the duplication of data where studies (or study populations) had multiple publications, the most recent and complete report was used as the main reference, but additional details were extracted from the other publications as necessary. Data were extracted from included studies by one reviewer and checked by a second reviewer.

### Data synthesis

Following data extraction, all data were initially tabulated and subsequently reported descriptively to answer the systematic review key questions regarding efficacy and safety of allo-HSCT.

### Risk of bias assessment

The quality of each individual study was assessed in order to ensure that the conclusions and findings of this review were based on the best available evidence and that any potential sources of bias in the data were identified.

No randomised control trials (RCT) were identified, therefore, study quality was assessed using the Joanna Briggs Institute (JBI) Critical Appraisal Checklist for Quasi-experimental Studies tool [30]. Two reviewers independently assessed the study quality and any discrepancies were resolved through discussion or the intervention of a third reviewer.

## Results

**Fig. 1:**
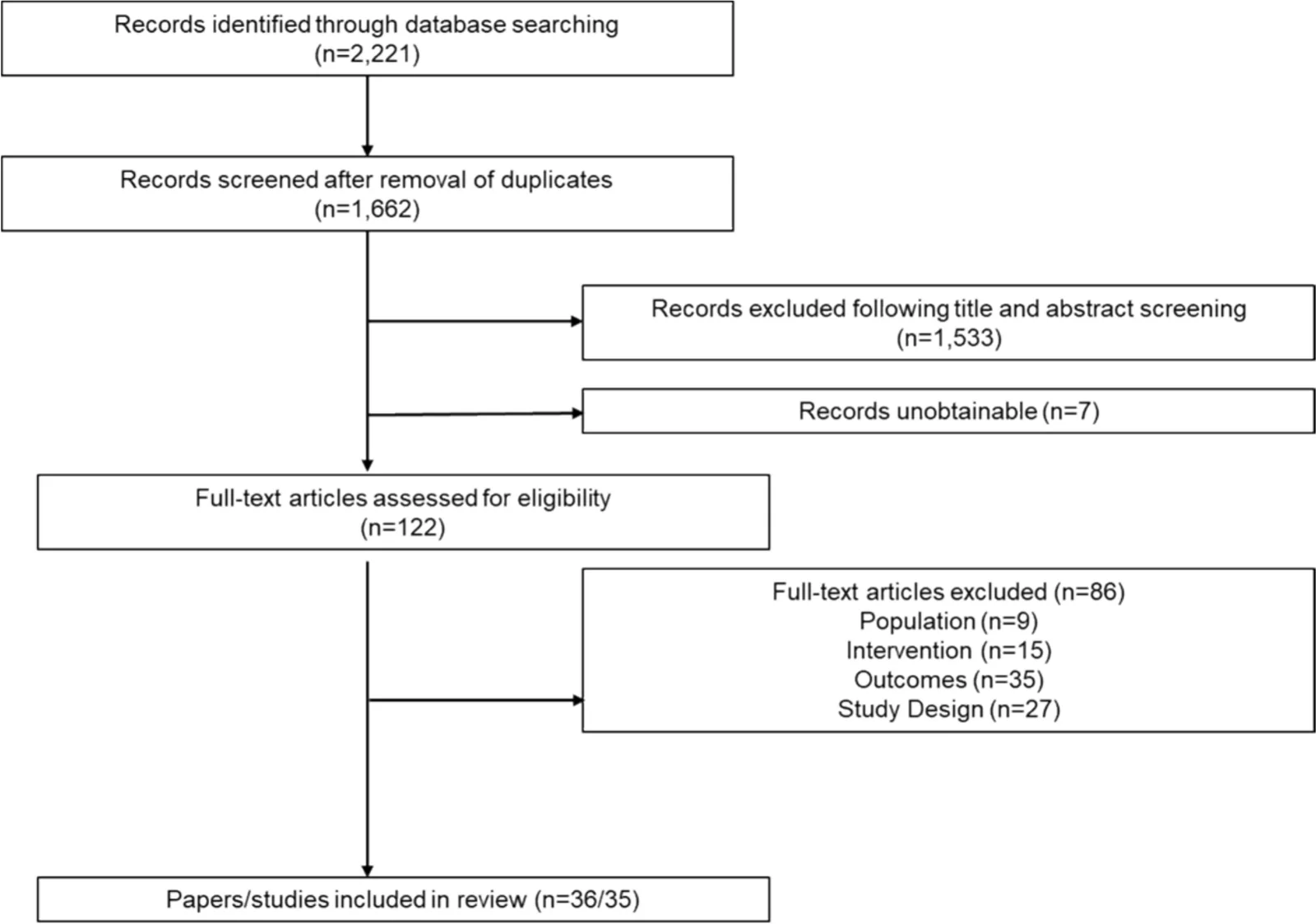
Study flowchart

Database searches returned 1,662 citations following the removal of duplications. One hundred and twenty-two papers underwent full text scrutiny against the inclusion/exclusion criteria. Following this, a total of 35 studies in 36 publications remained and were included within the review (Fig. 1).

### Study details

Table 1 shows characteristics of the 35 included studies, which were published between 2002 [31] and 2024 [24, 32]. Twenty-eight studies were retrospective non-comparative, five were prospective non-comparative and two were retrospective comparative. Twenty-four studies were reported as full papers, and 11 studies were reported in 12 conference abstracts (Borovkova [33] and Kirgizov [34] reported identical data). Median follow-up across studies ranged from 0.7 [35] to 14.3 [36] years. Two studies [37, 38] only reported mean follow-up times, of 2.6 and 6.4 years. Median follow-up was not reported in eight studies. Maximum follow-up across studies ranged from 1.5 [35] to 29 [16] years; nine studies did not report maximum follow-up times. Thirty studies had allo-HSCT arms, three studies allo-HSCT + ERT arms, and two studies did not report whether ERT was included.

**Table 1:** Study details of the included papers, including design, treatment arm and study follow-up

References	Study design	Study format	Median duration of study follow-up (years)	Maximum duration of study follow-up (years)	Arm name
Aldenhoven et al. [44]	Retrospective non-comparative	Conference abstract	NR	NR	HSCT
Aldenhoven et al. [47]	Retrospective non-comparative	Full paper	9.2	23	HSCT
Bjoraker et al. [45]	Prospective non-comparative	Full paper	5.6	21	HSCT
Bleeke et al. [37]	Retrospective non-comparative	Conference abstract	Mean = 2.6	8.3	HSCT
Boelens et al. [39]	Retrospective non-comparative	Full paper	4.8	6	HSCT
Borovkova et al. [33] and Kirgizov et al. [34]*	Retrospective non-comparative	Conference abstract	5	13.3	HSCT
Braunlin et al. [36]	Retrospective comparative	Full paper	14.3	28.4	HSCT
Bykova et al. [52]	Retrospective non-comparative	Full paper	2.8	10.2	HSCT
Cox-Brinkman et al. [35]	Retrospective comparative	Full paper	0.7	1.5	HSCT + ERT
De Souza et al. [53]	Retrospective non-comparative	Conference abstract	5	NR	NR
Dunseath et al. [59]	Retrospective non-comparative	Full paper	NR	NR	HSCT
Furlan et al. [48]	Retrospective non-comparative	Conference abstract	7.2	9.8	HSCT
Gardin et al. [23]	Retrospective non-comparative	Full paper	9	16.5	HSCT
Ghosh et al. [54]	Retrospective non-comparative	Full paper	3.8	10.3	HSCT + ERT
Grewal et al. [31]	Retrospective non-comparative	Full paper	2.1	3.8	HSCT
Guffon et al. [21]	Retrospective non-comparative	Full paper	20	33	HSCT
Hartmann et al. [42]	Retrospective non-comparative	Conference abstract	11.5	13.6	HSCT
Huang et al. [32]	Retrospective non-comparative	Full paper	NR	7	HSCT Surgery group
Kirgizov et al. [55]	Retrospective non-comparative	Conference abstract	4.2	11.7	HSCT
Lipinski et al. [24]	Retrospective non-comparative	Full paper	5.5	9.5	HSCT
Lum et al. [56]	Retrospective non-comparative	Full paper	NR	NR	HSCT
Maier et al. [46]	Prospective non-comparative	Full paper	8.1	16.1	HSCT
Pristanskova et al. [60]	Retrospective non-comparative	Conference abstract	3.8	14.3	HSCT
Pristanskova et al. [57]	Retrospective non-comparative	Full paper	NR	14.8	HSCT
Rodgers et al. [16]	Retrospective non-comparative	Full paper	10.7	29	HSCT
Schmidt et al. [38]	Retrospective non-comparative	Full paper	Mean = 6.4	22.5	HSCT
Shapiro et al. [49]	Retrospective non-comparative	Conference abstract	≥ 7	19.6	HSCT
Shapiro et al. [63]	Prospective non-comparative	Full paper	11.2	NR	HSCT
Souillet et al. [58]	Retrospective non-comparative	Full paper	4.7	16	HSCT
Svyatoslavovna Borovkova et al. [41]	Retrospective non-comparative	Conference abstract	1.5	3.7	HSCT
Taylor et al. [50]	Retrospective non-comparative	Full paper	8.5	19.2	HSCT
Tear Fahnehjelm et al. [43]	Prospective non-comparative	Full paper	12	17.4	HSCT
Thawrani et al. [51]	Retrospective non-comparative	Full paper	NR	NR	HSCT
Van Den Broek et al. [40]	Prospective non-comparative	Conference abstract	NR	NR	NR
Wynn et al. [64]	Retrospective non-comparative	Full paper	NR	NR	HSCT + ERT

*ERT* enzyme replacement therapy; *HSCT* haemopoietic stem cell transplantation; *NR* not reported

*These are two conference abstracts and report identical data

### Baseline characteristics

Table 2 describes characteristics of participants from the included studies. The total number of participants across studies was 1,831, and sample sizes ranged from four [24] to 258 [39]. The median age of participants at allo-HSCT ranged from 8 [40] to 29 [41] months. The percentage of male participants ranged from 20% [42] to 75% [43]. Fourteen studies did not report information of male percentage. Only three studies reported percentage of White Caucasian participants, which ranged from 91 to 100%.

**Table 2:** Baseline characteristics including number of participants, age, sex and ethnicity

References	Number of participants	Age on HSCT (months)	Sex (% of males)	Ethnicity (% White Caucasian, black)
Aldenhoven et al. [44]	197	Median 16	NR	NR
Aldenhoven et al. [47]	217	Median 16	56	91,9
Bjoraker et al. [45]	41	Mean 21.7	61	NR
Bleeke et al. [37]	19	Median 20.4	NR	NR
Boelens et al. [39]	258	Median 16.7	52	NR
Borovkova et al. [33]	38	Median 24	NR	NR
Braunlin et al. [36]	20 (with metabolic data)	Mean 18.7	75	NR
	43 (without metabolic data)	Mean 21.3	60	NR
Bykova et al. [52]	28	Median 24	39	NR
Cox-Brinkman et al. [35]	22 (HSCT + ERT)	Median 18	NR	NR
	142 (historic control)	Median 18	NR	NR
De Souza et al. [53]	8	NR	50	NR
Dunseath et al. [59]	25	Median 12.8	68	NR
Furlan et al. [48]	11	Median 19.6	NR	NR
Gardin et al. [23]	51	Median 18	NR	NR
Ghosh et al. [54]	81	Median 13.1	53	NR
Grewal et al. [31]	11	Median 25	36	NR
Guffon et al. [21]	25	Median 21	40	92,8
Hartmann et al. [42]	5	Median 14.4	20	NR
Huang et al. [32]	50	Mean 15.6	44	NR
Kirgizov et al. [55]	33	Median 15	NR	NR
Lipinski et al. [24]	4	NR	NR	NR
Lum et al. [56]	54	Median 15.1	53.7	NR
Maier et al. [46]	14	Mean 20.4	43	100
Pristanskova et al. [60]	32	Median 24.2	NR	NR
Pristanskova et al. [57]	35	Median 25.5	66	NR
Rodgers et al. [16]	134	Mean 21.8	51.5	NR
Schmidt et al. [38]	19	Mean 20.4	47.3	NR
Shapiro et al. [49]	10	Median 13.2	40	NR
Shapiro et al. [63]	7	Mean 12.6	57	NR
Souillet et al. [58]	27	Median 25	44	NR
Svyatoslavovna Borovkova et al. [41]	7	Median 29	NR	NR
Taylor et al. [50]	23	Mean 9.4	NR	NR
Tear Fahnehjelm et al. [43]	8	Median 12.2	75	NR
Thawrani et al. [51]	51	Mean 19.2	56.8	NR
Van Den Broek et al. [40]	63	Median 8	NR	NR
Wynn et al. [64]	18	Mean 12	55.5	NR

### Efficacy

Twenty seven studies were included that had > 5-years of follow-up efficacy data: seven [23, 40, 42–46] (with a total of 379 patients) evaluated effectiveness with continuous data (see Table 3); 10 studies [21, 23, 38, 42, 43, 47–51] (with a total of 907 patients) with categorical data (see Table 4); and 14 studies [16, 23, 24, 33, 39, 48, 52–58] (with a total of 800 patients) with time-to-event data (see Table 5). Eight studies were only included for safety data (i.e., no efficacy data with a follow-up of at least 5 years). [31, 32, 35, 37, 52, 55, 59, 60]

**Table 3:** Efficacy results from continuous data

Author	Outcome type	Outcome details	Units	Follow-up	N	Outcome at follow-up /change from baseline	Mean (SD or 95% CI)	Median (min–max/IQR)
Aldenhoven et al. [44]	Biochemistry	Normal enzyme levels (according to the	%	Median: 7.3 local reference range)	197	NR	NR	NR
Bjoraker et al. [45]	Daily living skills	Vineland Adaptive Behaviour Scales	NR	Fixed: 4 to 8	41	FU	58.1 (26.7)	NR
	Communication						63.5 (22.1)	NR
	Socialisation						75.4 (22.9)	NR
Gardin et al. [23]	Cognitive function	Age at first sentences in years	months	Median: 9	51		NR	3.5 (3,4)
	Psychomotor (gross and fine motor function)	Age at walking in months						18 (17,24)
	Skeletal abnormality	Final height	cm					140 (134,146)
		Height	SD					− 2.6 (− 4.4,− 1.3)
Hartmann et al. [42]	Skeletal abnormality	Head circumference	SDS	Median: 11.5	5	Change from baseline	− 0.17	NR
Maier et al. [46]	Skeletal abnormality	Body length		Median: 8.1	14	FU	− 1.61 (− 4.58,3.29)	NR
		Head circumference					0.91 (− 2.52,6.09)	NR
		Leg length					− 1.64 (− 3.88,1.49)	NR
		Sitting height					− 3.28 (− 7.37,0.26)	NR
		Weight					− 0.56 (− 3.19,2.95)	NR
Tear Fahnehjelm et al. [43]	Sensory function—vision	BCVA—left eye	Decimal	Median: 12	8		NR	0.5 (0.1–0.65)
		BCVA—right eye						0.4 (0.1–0.8)
		Central corneal thickness -left eye	μm					632 (547–627)
		Central corneal thickness—right eye						627 (541–795)
		High hyperopia—left eye	Dioptre					5.9 (4–8.75)
		High hyperopia—right eye						6 (4–7.75)
		Intraocular pressure	mm Hg		6	NR		NR
Van Den Broek et al. [40]	QoL	Physical functioning	z-score	Median: 8	63		− 2.4 (− 3.5, − 1.6)	NR
		Global functioning					− 3.2 (− 4.8, − 1.8)	NR
		Psychosocial health					0.15 (− 7, 8)	NR

*ADL* activities of daily living, *BCVA* best corrected visual activity, *CI* confidence intervals, *cm* centimetres, *FU* follow-up, *IQR* interquartile range, *max* maximum, *min* minimum, *Hg* millimetres of mercury, *NR* not reported, *QoL* quality of life, *μm* micrometre, *SD* standard deviation, *SDS* standard deviation scores

**Table 4:** Efficacy results for categorical data

Author	Outcome type	Outcome details	Follow-up type	Follow-up (years)	Total no. assessed (N)	% with outcome
Aldenhoven et al. [47]	Cardiovascular function	Cardiac arrest	Median	15.1	217	2.7
		Mitral valve insufficiency (progression)				37
	Pulmonary function	Tracheotomy for prolonged respiratory support		19.7		0.46
	Sensory function—vision	Progression of corneal clouding, resulting in corneal transplantation		11.1		9.8
	Sleep apnoea and adeno-tonsillar surgery	Overnight continuous positive airway pressure		7.8		3.6
	Cognitive function	Normal IQ (≥ 85)	Fixed	“Last”		26.9
		Mild impairment (55–70)				28.3
		Moderate/severe (≤ 70)				44.9
Furlan et al. [48]	Cognitive function	IQ^a^ improved from 64 to 85	Fixed	≥ 4	7	14
		IQ^a^ stabilised range 79–85				43
		IQ^a^ worsened range 41–57				43
	Sensory function—hearing	Improvement from mixed deafness				85.7
	Skeletal abnormality	Dorsolumbar kyphosis, hip dysplasia, genu valgum				100
Gardin et al. [23]	ADL including	Medically supervised full-time institution	Median	9	43	16
		Normal or remedial school (for ≥ 6 years old)				58
		Specialised school (for intellectual disability)				26
	Cardiovascular function	High blood pressure			47	9
		Hypertrophic cardiomyopathy				4
		Valvular disease (regurgitation/stenosis)^b^				72
	Cognitive function	50 ≤ IQ < 70			23	17
		70 ≤ IQ < 85				52
		Acquisition of an intelligible language			46	91
		Acquisition of reading/writing at 8 years of age			41	78
		IQ < 50 or not evaluable			23	13
		IQ ≥ 85				17
	Psychomotor (gross and fine motor function)	Complete loss of walking or permanent wheelchair use			46	15
		Fatigability with low walking perimeter				30
		No functional impairment				33
		Walking with support (cane or intermittent wheelchair)				22
	Pulmonary function	Reduction of lung volume in PFT (≤ 60% of normal)			30	47
	Sensory function—hearing and vision	Corneal clouding			47	96
		Corneal graft				15
		Hearing loss				74
		Retinal dystrophy (cone-rod dystrophy)			44	11
		VA impairment (before corneal graft) ≥ 8/10			36	42
		VA impairment (before corneal graft) 5/10 < VA < 8/10				11
		VA impairment (before corneal graft) VA ≤ 5/10				47
	Skeletal abnormality	Surgery for cervical cord C1-C2 compression			47	13
	Sleep apnoea and adeno-tonsillar surgery	Use of non-invasive ventilation			45	20
	Socialisation	Adapted employment (for mental disability)			13	23
		Good socio-professional insertion				62
Guffon et al. [21]	Cardiovascular function	Valve stenosis	Fixed	7	25	24
	Cognitive function	Severe cognitive impairment (IQ less than 40)				20
		Wechsler intelligence scale (overall lower scores on both WMI (median WMI = 69.5; n = 16) and PSI (median PSI = 65)				64
	Pulmonary function	Normal FVC > 80%				12
		Restrictive syndrome				60
	Sensory function—hearing and vision	Cataract				16
		Corneal clouding				100
		Sensorineural hearing impairment				72
	Skeletal abnormality	Hip dysplasia				100
		Scoliosis				96
Hartmann et al. [42]	Cognitive function	Normal global IQ	Median	11.5	5	40
	Communication	No expressive language				20
		Speech development without impairment				40
	Sensory function—hearing and vision	Corneal clouding				100
		Corneal transplantation				20
		Impaired sensorineural hearing				40
	Skeletal abnormality	Kyphoscoliosis				100
		Unable to walk				60
Schmidt et al. [38]	Skeletal abnormality	Scoliosis	Mean	6.4	19	63
		Thoracolumbar gibbus				89
Shapiro et al. [49]	Psychomotor (gross and fine motor function)	Spinal cord compression	Fixed	≥ 7	10	0
	Sensory function—vision	Corneal clouding				100
Taylor et al. [50]	Skeletal abnormality	Genu valgum	Mean	8.5	23	52.1
		Hip dysplasia				100
Tear Fahnehjelm et al. [43]	Sensory function—vision	Corneal opacity	Median	12	8	100
		High hyperopia				100
		Increased corneal clouding (since HSCT)				62.5
		Photophobia				75
		Poor night vision				75
		Poor VA				75
		Retinal degeneration				12.5
Thawrani et al. [51]	Skeletal abnormality	Acetabular dysplasia	Fixed	4.5	51	100
		Proximal femur valgus				100

*ADL* activities of daily living; *FVC* Forced Vital Capacity; *HSCT* haemopoietic stem cell transplantation; *IQ* intelligence quotient; *PFT* pulmonary function tests; *PSI* processing speed index; *VA* visual acuity; *WMI* working memory index

^a^Before HCT IQ ranged from 64 to 93

^b^Mitral (97%) and/or aortic (38%)

**Table 5:** Efficacy data for time to event data including OS and EFS

Author	Outcome details	Follow-up type	Follow-up (years)	Total no. assessed (N)	% with outcome
Boelens et al. [39]	OS	Fixed	5	258	74
	EFS^a^				63
Borovkova et al. [33]	OS	Median		38	87
Bykova et al. [52]	OS	Fixed		28	89
	EFS^b^				57
De Souza et al. [53]	OS	Median	5	8	37.5
Furlan et al. [48]			7.2	11	91
Gardin et al. [23]			9	51	92.1
Ghosh et al. [54]	OS		3.8	81	86
	EFS^c^				80
Kirgizov et al. [55]	OS	Fixed	11.7	33	82
Lipinski et al. [24]		Median	5.5	4	100
Lum et al. [56]		Fixed	20	54	73.7
Pristanskova et al. [57]		Median	NR (up to 14.8 years on K-M curve)	35	82
Rodgers et al. [16]		Fixed	10	134	62
			25		37
Souillet et al. [58]		Median	4.7	27	85

*EFS* event-free survival, *K-M* Kaplan–Meier, *NR* not reported, *OS* overall survival

^a^Graft failure (defined as a lack of neutrophil recovery or transient engraftment of donor cells after transplantation and/or a requirement for a second transplant), or death

^b^Any adverse event (death, primary graft failure or rejection, grade III–IV aGvHD, or widespread chronic GVHD)

^c^Event NR

#### IDUA enzyme activity

Only one study reported long-term IDUA enzyme activity: Aldenhoven et al. [44] reported that 75% of patients achieved enzyme activity within the normal range at a median of 7.3 years post-allo-HSCT.

#### Neurocognitive and communication outcomes

Neurocognitive and communication outcomes showed considerable heterogeneity. Gardin et al. [23] reported that at a median follow-up of 9 years 91% of transplanted patients developed intelligible speech and 78% achieved literacy by age 8, although IQ distributions varied widely, with only 17% in the average range [45]. Aldenhoven [47] reported that only 26.9% had a normal IQ at the last follow-up. Furlan et al. [48] demonstrated that over half of patients showed stable or improved IQ following allo-HSCT, but from a relatively low baseline (before allo-HSCT IQ ranged from 64 to 93). Note that, although only reported in one study by Aldenhoven [47], IQ was higher if allo-HSCT was performed earlier, the coefficient for a regression analysis being significant for age at treatment.

#### Psychomotor outcomes

Psychomotor outcomes were similarly variable. It was reported by Gardin et al. [23], that one-third of patients remained free of functional impairment in ambulation at a median of nine years follow-up, while 15% were permanently wheelchair-dependent. According to Hartmann 2017, as many as 60% were unable to walk at a median of 11.5 years follow-up [42].

#### Skeletal manifestations

Skeletal manifestations remained largely refractory. Several studies documented near-universal prevalence of hip dysplasia, kyphosis, and scoliosis, often necessitating surgical interventions despite otherwise successful transplantation [21, 38, 51]. As with IQ, Aldenhoven [47] also showed that cord compression and carpal tunnel syndrome were significantly less likely with earlier allo-HSCT.

#### Ophthalmological and auditory complications

Ophthalmological complications were frequent. Tear Fahnehjelm et al. [43] reported corneal opacity in all patients and progressive visual impairment in the majority. Aldenhoven et al. [47] noted progression of corneal clouding sufficient to require transplantation in 10% of patients at a median follow-up of 11.1 years. Gardin et al. [23] found almost universal corneal clouding and 42% with significant visual impairment at a median follow-up of 9 years, and both Guffon 2021 [21] and Shapiro 2009 [49] reported 100% corneal clouding at 7 years follow-up. It can be difficult to know whether corneal problems arise because of the condition or can be attributed to the treatment. However, Gardin et al. [23] also stated that “progressive corneal clouding” post-alloHSCT was observed, and Tear Fahnehjelm [43] reported that 62.5% had an increase in corneal clouding post-allo-HSCT. Hearing loss also remained common, affecting 40% (median follow-up 11.5 years) in one very small study (n = 5) [42]. In two larger studies, 72% at 7 years, and 74% (median follow-up 9 years) had some hearing loss [21, 23].

#### Cardiovascular and pulmonary involvement

Cardiovascular and pulmonary involvement persisted in a substantial proportion of patients. Aldenhoven et al. [47] reported progression of mitral valve disease in more than one-third of patients (37% of 217 patients) over a median follow-up of 15.1 years. Gardin et al. [23] described mitral involvement in 97% and aortic disease in 38% of transplanted survivors. Restrictive lung disease was reported in 47–60% of patients in long-term follow-up cohorts [21, 23]. Aldenhoven [47] also showed that mitral and aortic valve insufficiency was significantly less likely with earlier allo-HSCT.

#### Survival

In studies where median follow-up was reported (between 3.8 and 9 years), OS was at least 80% in all but one study [23, 24, 33, 48, 52–54, 58]. In studies reported OS at a fixed timepoint, five-year OS was 74% and 89% in Boelens 2013 [39] and Bykova 2020 [52]. Longer-term follow-up was reported in two studies: Rodgers et al. [16] reported OS of 62% at 10 years and 37% at 25 years, and Lum 2017 reported 73.7% survival at 20 years [56]. Five-year event-free survival (EFS) ranged from 57 to 63% in Bykova 2020 [52] and Boelens 2013 [39] respectively. However, the definition of an event varied or was not reported [39, 52, 54]. Note that, although it was only reported in one study, by Boelens [39] EFS was higher if HSCT was conducted earlier: 71% vs. 55% for age at transplant less than vs. more than the median of 16.7 months, respectively.

### Safety

Nineteen studies, including a total of 849 patients, reported safety outcomes.

#### Acute graft-versus-host disease

Acute graft-versus-host disease (GvHD) (grade II–IV) occurred in 17–57% of patients [41, 54] with severe grade III–IV disease in up to 28%. Chronic GvHD occurred in 0–36% of cases [33, 37, 52], although extensive disease was less common (≤ 7%).

#### Infectious complications

Infectious complications were frequent, with Dunseath et al. [59] reporting bloodstream infections in 48% and Borovkova et al. [33] describing pulmonary infections in 30%. Mortality related to GvHD, infections, or other causes in studies with a follow-up up to five years ranged from 9% (only 1 patient) to 13.1% [33, 48, 54]. At 25 years, mortality rose to 63% in Rodgers et al*.* [16] Rodgers et al*.* [16] also showed that in the short-term, infection was the most common cause, and in the long-term it was pulmonary-related (See Table 6).

**Table 6:** Safety results

Author	Outcome	Severity	Follow-up type	Follow-up (years)	Total no. assessed (N)	% with outcome
Bleeke et al. [37]	Acute GvHD	Grade II–IV	Fixed	8.3	19	37
		Grade III–IV				10.5
	Chronic GvHD	All grades				0
Boelens et al. [39]	Acute GvHD	Grade II		0.3	258	18
		Grade III				3
		Grade IV				4
		Grade II–IV				25
	Chronic GvHD	All grades		5		16
Borovkova et al. [33]	Acute GvHD	Grade II	Median		38	37
		Grade III–IV				5
	Chronic GvHD	Extensive				0
		Local				8
	Early severe toxicity	NR				0
	Late effects			1.3		47.4
	Pulmonary infection			5		30
	pulmonary infection late effect	Serious		1.3		8
	Death	NR			38	13.1 (caused by: 7.9 infections, 2.6 Transfusion-Related Acute Lung injury, 2.6 acute GvHD)
Bykova et al. [52]	Acute GvHD	Grade II–IV		2.8	28	43
		Grade III–IV				18
	Chronic GvHD	Extensive				7
		Local				29
de Souza et al. [53]	Infections	NR		5	8	25
Dunseath et al. [59]	GvHD	All grades	NR	NR	25	24
		Grade I–II				16
		Grade III–IV				8
	Bloodstream infections	NR				48
Furlan et al. [48]	Death		Fixed	1	11	9 (caused by following immunological complications)
Ghosh et al. [54]	Acute GvHD	Grade II–IV	Median	3.8	81	17
		Grade III–IV				4
	Chronic GvHD	NR				11
	Death					12 (caused by: 7.2 infections, 2.4 GvHD, 1.2 veno-occlusive disease, 1.2 unknown)
Grewal et al. [31]	Acute GvHD	All grades		2.1	11	100
		Grade I				55
		Grade II–IV				45
		Grade III–IV				27
Huang et al. [32]	Transplant-related complications	NR	Fixed	9.9	54	18.51
Kirgizov et al. [55]	Acute GvHD	Grade II	Median	4.2	33	42
		Grade III–IV				6
	Chronic GvHD	Extensive				0
		Local				9
	Severe toxicity	NR				0
	Infections—three patients, Transfusion-related acute lung injury—one patient, Acute GvHD—one patient	NR				13.1
	Pulmonary infection					30
Maier et al. [46]	Acute GvHD	Grade I	Fixed	8.1	14	21.4
	Chronic GvHD	All grades				7.1
Pristanskova et al. [57]	Acute GvHD	Grade I–II		14.8	38	29
		Grade III–IV				8
	Chronic GvHD	All grades				8
Rodgers et al. [16]	Death	NR		25	134	63 (caused by: 12 infections, 8 cardiac related, 27 pulmonary-related, 16 other complications-related)
Shapiro et al. [63]	GvHD	Mild degree	Mean	11.2	7	14.2
Souillet et al. [58]	Acute GvHD	Grade I–II	Median	4.7	27	18.5
Svyatoslavovna Borovkova et al. [41]		Grade II		1.5	7	29
		Grade III				14
		Grade IV				14
		Grade II–IV				57
		Grade III–IV				28
	Severe toxicity	Class III–IV				0
Wynn et al. [64]	GvHD	NR	Fixed	≥ 0.5	18	5.5
	Viral infection					5.5

*GvHD* graft versus host disease, *NR* not reported

### Risk of bias quality assessment

Risk of bias was assed using the JBI Critical Appraisal Tool for quasi-experimental studies. Overall, study quality was variable, with most studies judged to have a moderate risk of bias. Across the 35 included studies, only two studies met nearly all the criteria, demonstrating a low risk of bias [32, 36]. Most studies clearly described the causal relationship and measured outcomes consistently, but important limitations were common. The absence of control groups was universal, and multiple pre- and post-intervention measurements were infrequently reported. Incomplete follow-up was noted in several studies, and differences between groups were often not adequately addressed. Outcome measurement methods were generally reliable, but reporting was frequently unclear for statistical analyses and handling of missing data. Abstract-only publications lacked sufficient methodological detail to permit robust assessment and were judged at high risk of bias [40, 53]. Full reporting of the risk of bias quality assessment is provided in Additional file 1.

## Discussion

This systematic review consolidates outcomes from 35 studies evaluating the long-term efficacy and safety of allo-HSCT, with or without the combination of ERT, in patients with MPS-IH. Collectively, the data support allo-HSCT as the standard of care for eligible infants, demonstrating clear survival and neurocognitive benefits. Nevertheless, residual somatic disease, variable long-term outcomes, and procedure-related risks remain major challenges.

### Efficacy

This systematic review demonstrates that allo-HSCT significantly alters the natural history of MPS-IH. Without intervention, survival is limited to the first decade of life, with UK registry data reporting a median (50%) survival of 8.7 years, reduced to 6.8 years when patients were censored for transplantation [9]. Against this background, the survival benefit of allo-HSCT seems substantial. Across studies reporting median follow-up (between 3.8 and 9 years), OS was at least 80% in most studies [24, 33, 52, 54]. Long-term follow-up confirmed survival exceeding 50%, including up to 62% at 10 years in one study, and 73.7% at 20 years in another [16, 56]. Event-free survival was lower, ranging between 57 and 80% at 3–5 years, [39, 52, 54] reflecting the impact of graft failure and disease progression, yet still indicating durable benefit for the majority of patients.

The principal advantage of allo-HSCT is neurocognitive preservation, attributable to donor-derived enzyme delivery across the blood–brain barrier [61, 62]. Gardin et al. [23] reported that most transplanted children developed speech and literacy skills, and Furlan et al*.* [48] observed stabilisation or improvement of IQ in over half of patients. Nevertheless, persistent deficits in adaptive behaviour and executive function were described in several studies [21, 45], and outcomes varied considerably. The age at which allo-HSCT is performed did seem to be a key prognostic factor, with earlier transplantation associated with superior outcomes [39, 47]. Psychomotor outcomes showed similar heterogeneity, as some children retained normal ambulation while others experienced progressive decline despite engraftment [23, 42].

While allo-HSCT clearly improves survival and preserves neurological development relative to the untreated course, many somatic manifestations persist. Skeletal disease was almost universal, with progressive hip dysplasia, kyphosis, and scoliosis frequently requiring surgical intervention [21, 38, 51]. Ophthalmological outcomes were similarly poor, with corneal opacity and visual impairment affecting most survivors [23, 43]. Hearing loss was present in up to about three-quarters of patients [21, 42]. Cardiovascular involvement was common, with valvular disease reported in over one-third of transplanted patients [23, 47], and restrictive pulmonary disease occurred in nearly half of long-term survivors [21]. These results demonstrate that while allo-HSCT alters prognosis and preserves critical functions, it does not fully correct multisystem pathology. Furthermore, these complications necessitate ongoing respiratory and cardiology follow-up in the long term.

### Safety

Allo-HSCT for MPS-IH remains associated with considerable risks, although outcomes have improved substantially over time. Acute GvHD occurred in up to 57% of patients, with severe grade III–IV disease reported in up to 28% [41, 54]. Chronic GvHD was observed in up to 36% [33, 37, 52], although extensive forms were less common. Infections were also a frequent complication [33, 59]. These complications, particularly infection, contributed to mortality earlier on (first five years) in analysed cohorts [33, 54]. However, other causes of death, especially pulmonary-related, seem to be much more common in the long term (25 years follow-up) [16]. Nevertheless, the frequency of serious complications underscores the need for careful patient selection and management in specialist centres.

### Limitations and future research

The evidence base for allo-HSCT in MPS-IH is limited by several factors that warrant caution in interpretation. Firstly, the majority of included studies were retrospective and observational, with inherent risks of bias and incomplete data capture. Prospective data were rare and often confined to small single-centre cohorts, limiting generalisability. Secondly, sample sizes across studies were small, reflecting the rarity of MPS-IH, and there was also much variation in age at transplantation and the median follow-up duration of the included studies. As a result, long-term outcomes such as late-onset cardiopulmonary complications or survival beyond 20 years remain uncertain. A further limitation is that outcome measures, particularly neurocognitive and adaptive functioning assessments, were heterogeneous. Different centres employed diverse psychometric tools, precluding robust cross-study comparison and complicating pooled interpretation.

Another important consideration in the interpretation of the long-term outcomes for HSCT is the timing of the included studies. The inclusion criteria for this systematic review were broad and consequently outcomes from older studies are contributing to the evidence base, which could impact conclusions regarding both clinical and safety outcomes as some of the poorer outcomes realised with HSCT come from the early transplant era. There have been significant advances in transplantation strategies in recent years to overcome some of the known limitations with allo-HSCT and improve efficacy and safety outcomes. For example, improved supportive care including the use of ERT; better donor selection; cord blood transplantation; less toxic conditioning regimens such as the use of treosulfan-based conditioning or pharmacokinetic-guided busulfan; and improved GvHD prophylaxis. An interesting future improvement to this systematic review, if more data were to become available, would be to stratify and compare clinical outcomes by transplant era to quantify this.

Consequently, all of the aforementioned heterogeneity across the included studies may confound apparent differences in efficacy or safety. An additional limitation is reporting bias cannot be excluded, successful transplants are more likely to be reported by high-volume centres, while negative or complicated cases may be underrepresented. The present review is also limited to published data; registry data may contain further insights that are not yet in the public domain. In relation to this, the authors acknowledge that there might be overlap in patient cohorts between studies, as suggested by overlap in authorship and institutions. However, all studies had to be treated as unique if their sample sizes and baseline characteristics were different, and there was no explicit indication of connection. Finally, the methodological quality of the included studies was generally moderate with few judged at low risk of bias. Most were observational cohorts without control groups, with incomplete follow-up and limited reporting of statistical methods, which reduces the certainty of the evidence. Future research should prioritise prospective, multicentre studies that employ harmonised protocols for outcome measurement, particularly for neurocognitive and functional assessments. The development of international consensus on standardised endpoints would allow focused comparison across cohorts. Greater focus on long-term follow-up is also required to better understand survival beyond two decades, late effects of conditioning, and the durability of engraftment. Adjunctive strategies, such as peri-transplant enzyme replacement therapy, require rigorous evaluation to determine their role in improving somatic outcomes.

Furthermore, emerging therapeutic strategies, such as autologous gene-modified HSCT, intrathecal or brain-penetrant enzyme delivery, and systemic gene therapy, are being developed to address the persistent disease manifestations that remain insufficiently corrected by conventional allo-HSCT. For example, results from a phase I/II study of an autologous gene-modified HSCT therapy that utilizes a lentiviral vector (LVV) to deliver a functional copy of the IDUA gene into the patient’s cells showed notable improvements in in skeletal, ocular, and cardiac outcomes in comparison to an age-matched retrospective allo-HSCT cohort [25–27]. Future results from an ongoing, prospective, randomised-controlled trial of this autologous gene-modified HSCT (OTL-203) vs. allo-HSCT will be useful to confirm these findings [NCT06149403]. In addition, a phase I/II study of another type of gene therapy utilizing an adeno-associated virus serotype 9 (AAV9) carrying the IDUA gene delivered directly into the cerebrospinal fluid is under way for the treatment of MPS-IH [NCT03580083]. These approaches are hypothesised to provide enhanced efficacy by achieving both supranormal and more sustained IDUA enzyme activity, as well as improved biodistribution to traditionally hard-to-reach or immune-privileged sites, including the brain and eyes, where allo-HSCT has limited penetration. Continued development and rigorous evaluation of these modalities, supported by large collaborative international registries, will be essential to generate real-world evidence on their effectiveness, safety, and long-term durability [26].

## Conclusion

In conclusion, allo-HSCT appears to be an effective intervention for MPS-IH that can meaningfully extend survival and preserve neurological and physical function in the long-term, particularly when administered early. However, it is not curative across all disease domains, with skeletal, cardiovascular and sensory deficits often persisting, but it remains the standard of care in modifying the disease course. The procedure carries measurable risks, particularly related to mortality, GvHD and infection. Some of these are generally manageable with current supportive care protocols and have improved with the use of modern transplant strategies. Survival of approximately 80% at 5 years and 40% at 25 years is encouraging, but with severe late morbidity, so there remains an opportunity for improvement. Consequently, continued research and innovation are essential to improve long-term outcomes and quality of life for individuals with MPS‑IH.

## Supplementary Information

Additional file1 (DOCX 29 KB)

## Data Availability

The synthesized data and methods for this systematic review are presented in the manuscript text and tables.
