# Supplementary material for: Efficacy and safety of haemopoietic stem cell transplant (HSCT) for mucopolysaccharidosis type I-Hurler syndrome (MPS-IH): a systematic review
Source: Stem Cell Res Ther. 2026 Aug 12;17:415. doi: 10.1186/s13287-026-05155-6 (PMC13749707; doi:10.1186/s13287-026-05155-6)
Supplement: Supplementary file 1 [file 13287_2026_5155_MOESM1_ESM.docx]

## Literature searches

Literature searches were conducted to identify studies investigating the use of hematopoietic stem-cell transplantation (HSCT) in the treatment of mucopolysaccharidosis Type IH (MPS-IH). The search strategies were designed using the recommendations of the Centre for Reviews and Dissemination (CRD) guidance for undertaking reviews in health care{Centre for Reviews and Dissemination, 2009 [accessed 23.3.11] #2} and the Cochrane Handbook.{Higgins, 2023 [accessed 6.3.24] #1} Search strategies combined relevant search terms comprising indexed keywords (e.g. Medical Subject Headings, MeSH) and free text terms appearing in the titles and/or abstracts of bibliographic database records. Search terms were identified through discussion between the review team, by scanning background literature and ‘key articles’ already known to the project team, and by browsing database thesauri. The search strategies were developed specifically for each database and the keywords adapted according to the configuration of each database. Searches were not limited by study design, language, publication status (unpublished or published) or date of publication.

The following databases were searched from inception:

- MEDLINE and Epub Ahead of Print, In-Process, In-Data-Review & Other Non-Indexed Citations and Daily (Ovid): 1946 to December 16, 2024
- Embase (Ovid): 1974 to 2024 December 16
- Cochrane Central Register of Controlled Trials (CENTRAL) (Wiley Online Library): Issue 11 of 12, November 2024
- Cochrane Database of Systematic Reviews (CDSR) (Wiley): Issue 12 of 12, December 2024
- Northern Light Life Sciences Conference Abstracts (Ovid): 2010 - 2024 Week 49

Completed and ongoing trials were identified by searches of the following resources:

- ClinicalTrials.gov (<http://www.clinicaltrials.gov/>): to 17.12.24
- WHO International Clinical Trials Registry Platform (ICTRP) (<http://www.who.int/ictrp/en/>): to 17.12.24
- Orphanet (<https://www.orpha.net/>): to 17.12.24

Full search strategies are provided in Appendix 1.

### Reference checking

The bibliographies of identified research and review articles were checked for relevant studies.

### Handling of citations

Identified references from the bibliographic database searches were downloaded into EndNote bibliographic management software for further assessment and handling. Individual records within the EndNote libraries were tagged with searching information, such as searcher, date searched, database host, database searched, strategy name and iteration, theme or search question. This enabled the information specialist to track the origin of each individual database record, and its progress through the screening and review process.

### Quality assurance within the search process

For all searches undertaken by the Kleijnen Systematic Reviews Information team, the main Embase strategy was independently peer reviewed by a second KSR Information Specialist. Strategy peer review was informed by items based on the CADTH checklist.{Canadian Agency for Drugs and Technologies in Health, 2016 [accessed 16.11.20] #41}

**APPENDIX 1 – SEARCH STRATEGIES**

| **Resource** | **Host** | **Date range** | **Date searched** | **Records found** | **Records found after deduplication** |
| --- | --- | --- | --- | --- | --- |
| MEDLINE, Epub Ahead of Print, In-Process, In-Data-Review & Other Non-Indexed Citations, Daily | Ovid | 1946 to December 16, 2024 | 17.12.24 | 553 | 524 |
| Embase | Ovid | 1974 to 2024 December 16 | 17.12.24 | 1439 | 984 |
| CENTRAL | Wiley | Issue 11 of 12, November 2024 | 17.12.24 | 25 | 17 |
| CDSR | Wiley | Issue 12 of 12, December 2024 | 17.12.24 | 3 | 3 |
| Northern Light Life Sciences Conference Abstracts | Ovid | 2010 - 2024 Week 49 | 17.12.24 | 101 | 34 |
| ClinicalTrials.gov | <http://www.clinicaltrials.gov/> | to 17.12.24 | 17.12.24 | 57 | 57 |
| WHO ICTRP | <http://www.who.int/ictrp/en/> | to 17.12.24 | 17.12.24 | 13 | 13 |
| Orphanet | <https://www.orpha.net/en/research-trials/research-projects> | to 17.12.24 | 17.12.24 | 30 | 30 |
| **TOTAL** |  |  |  | **2221** | **1662** |

**Ovid MEDLINE(R) and Epub Ahead of Print, In-Process, In-Data-Review & Other Non-Indexed Citations, Daily and Versions (Ovid): 1946 to December 16, 2024**

**Date searched: 17.12.24**

**Records found: 553**

1	exp Mucopolysaccharidoses/	7210

2	((hurler$ or scheie$ or hurler-scheie$ or hurlers-scheie$ or hurlerscheie$ or hurlersscheie$ or pfaundler-hurler$ or Ullrich-Scheie$ or pfaundlerhurler$ or UllrichScheie$) adj3 (syndrome$ or disease$)).ti,ab,ot.	1180

3	(mucopolysaccharidos?s or muco-polysaccharidos?s or MPS$ or chondroosteodysplasia or chondroosteodystrophy or chondroosteoplasia or dysostosis multiplex or gargolylism$ or gargoylism$ or helmholtz harrington syndrome$ or iduronidase deficiency syndrome$ or lipochondrodystrophy or lipochondrodystrophies or mckusick 25280).ti,ab,ot.	27414

4	(geleophysic adj3 (dysplasia or dwarfism)).ti,ab,ot.	76

5	(Hunter$ adj3 (syndrome$ or disease$ or glossitis)).ti,ab,ot.	1198

6	McKusick 30990.ti,ab,ot.	0

7	(Maroteaux Lamy or polydystrophic dwarfism).ti,ab,ot.	361

8	(Morquio adj3 (syndrome$ or disease$)).ti,ab,ot.	589

9	((keratan or kerato) adj3 sulfaturia).ti,ab,ot.	4

10	(familial osseous dystrophy or keratosulfaturia or osteochondrodystrophia deformans).ti,ab,ot.	8

11	(Mucinos?s or mucinase or Mucinex or mucinol or mucinolytic).ti,ab,ot.	1447

12	(Sly adj3 (disease$ or syndrome$)).ti,ab,ot.	97

13	heparitinuria.ti,ab,ot.	0

14	((San Filippo or Sanfilippo$) adj3 (disease$ or syndrome$)).ti,ab,ot.	646

15	or/1-14	32109

16	exp Hematopoietic Stem Cell Transplantation/	61095

17	((h?ematopoietic stem cell$ or HSC or peripheral blood stem cell$) adj3 (transplant$ or therap$)).ti,ab,ot.	40133

18	(HSCT or HSCTs).ti,ab,ot.	18272

19	or/16-18	75628

**20	15 and 19	553**

**Embase (Ovid): 1974 to 2024 December 16**

**Date searched: 17.12.24**

**Records found: 1439**

1	exp mucopolysaccharidosis/ or ((hurler$ or scheie$ or hurler-scheie$ or hurlers-scheie$ or hurlerscheie$ or hurlersscheie$ or pfaundler-hurler$ or Ullrich-Scheie$ or pfaundlerhurler$ or UllrichScheie$) adj3 (syndrome$ or disease$)).ti,ab,ot.	13718

2	(mucopolysaccharidos?s or muco-polysaccharidos?s or MPS$ or chondroosteodysplasia or chondroosteodystrophy or chondroosteoplasia or dysostosis multiplex or gargolylism$ or gargoylism$ or helmholtz harrington syndrome$ or iduronidase deficiency syndrome$ or lipochondrodystrophy or lipochondrodystrophies or mckusick 25280).ti,ab,ot.	36771

3	(geleophysic adj3 (dysplasia or dwarfism)).ti,ab,ot.	102

4	(Hunter$ adj3 (syndrome$ or disease$ or glossitis)).ti,ab,ot.	1679

5	McKusick 30990.ti,ab,ot.	0

6	(Maroteaux Lamy or polydystrophic dwarfism).ti,ab,ot.	513

7	(Morquio adj3 (syndrome$ or disease$)).ti,ab,ot.	846

8	((keratan or kerato) adj3 sulfaturia).ti,ab,ot.	4

9	(familial osseous dystrophy or keratosulfaturia or osteochondrodystrophia deformans).ti,ab,ot.	5

10	(Mucinos?s or mucinase or Mucinex or mucinol or mucinolytic).ti,ab,ot.	1706

11	(Sly adj3 (disease$ or syndrome$)).ti,ab,ot.	141

12	heparitinuria.ti,ab,ot.	0

13	((San Filippo or Sanfilippo$) adj3 (disease$ or syndrome$)).ti,ab,ot.	956

14	or/1-13	43921

15	exp hematopoietic stem cell transplantation/ or ((h?ematopoietic stem cell$ or HSC or peripheral blood stem cell$) adj3 (transplant$ or therap$)).ti,ab,ot.	117302

16	(HSCT or HSCTs).ti,ab,ot.	42960

17	or/15-16	122440

**18	14 and 17	1439**

**The Cochrane Library (Wiley)**

[**https://www.cochranelibrary.com/**](https://www.cochranelibrary.com/)

**Cochrane Database of Systematic Reviews**

**Issue 12 of 12, December 2024**

**Date searched: 17.12.24**

**Records found: 3**

**Cochrane Central Register of Controlled Trials**

**Issue 11 of 12, November 2024**

**Date searched: 17.12.24**

**Records found: 25**

#1	MeSH descriptor: [Mucopolysaccharidoses] explode all trees	108

#2	((hurler* or scheie* or (hurler next scheie*) or (hurlers next scheie*) or hurlerscheie* or hurlersscheie* or (pfaundler next hurler*) or (Ullrich next Scheie*) or pfaundlerhurler$ or UllrichScheie*) NEAR/3 (syndrome* or disease*))	20

#3	mucopolysaccharidos?s or (muco next polysaccharidos?s) or MPS* or chondroosteodysplasia or chondroosteodystrophy or chondroosteoplasia or "dysostosis multiplex" or gargolylism* or gargoylism* or (helmholtz next harrington next syndrome*) or (iduronidase next deficiency next syndrome*) or lipochondrodystrophy or lipochondrodystrophies or "mckusick 25280"	1999

#4	geleophysic NEAR/3 (dysplasia or dwarfism)	0

#5	Hunter* NEAR/3 (syndrome* or disease* or glossitis)	86

#6	"McKusick 30990"	0

#7	"Maroteaux Lamy" or "polydystrophic dwarfism"	18

#8	Morquio NEAR/3 (syndrome* or disease*)	46

#9	(keratan or kerato) NEAR/3 sulfaturia	0

#10	"familial osseous dystrophy" or keratosulfaturia or "osteochondrodystrophia deformans"	0

#11	Mucinos?s or mucinase or Mucinex or mucinol or mucinolytic	26

#12	Sly NEAR/3 (disease* or syndrome*)	5

#13	heparitinuria	0

#14	("San Filippo" or Sanfilippo*) NEAR/3 (disease* or syndrome*)	30

#15	#1 or #2 or #3 or #4 or #5 or #6 or #7 or #8 or #9 or #10 or #11 or #12 or #13 or #14	2068

#16	MeSH descriptor: [Hematopoietic Stem Cell Transplantation] explode all trees	2572

#17	((h?ematopoietic next stem next cell*) or HSC or (peripheral next blood next stem next cell*)) NEAR/3 (transplant* or therap*)	6108

#18	HSCT or HSCTs	2273

#19	#16 or #17 or #18	6475

#20	#15 and #19	28

**#21	#20 in Cochrane Reviews, Cochrane Protocols	3**

**#22	#20 in Trials	25**

**Northern Light Life Sciences Conference Abstracts (Ovid): 2010 - 2024 Week 49**

**Date searched: 17.12.24**

**Records found: 101**

1	((hurler$ or scheie$ or hurler-scheie$ or hurlers-scheie$ or hurlerscheie$ or hurlersscheie$ or pfaundler-hurler$ or Ullrich-Scheie$ or pfaundlerhurler$ or UllrichScheie$) adj3 (syndrome$ or disease$)).ti,ab.	97

2	(mucopolysaccharidos?s or muco-polysaccharidos?s or MPS$ or chondroosteodysplasia or chondroosteodystrophy or chondroosteoplasia or dysostosis multiplex or gargolylism$ or gargoylism$ or helmholtz harrington syndrome$ or iduronidase deficiency syndrome$ or lipochondrodystrophy or lipochondrodystrophies or mckusick 25280).ti,ab.	3502

3	(geleophysic adj3 (dysplasia or dwarfism)).ti,ab.	12

4	(Hunter$ adj3 (syndrome$ or disease$ or glossitis)).ti,ab.	108

5	McKusick 30990.ti,ab.	0

6	(Maroteaux Lamy or polydystrophic dwarfism).ti,ab.	16

7	(Morquio adj3 (syndrome$ or disease$)).ti,ab.	38

8	((keratan or kerato) adj3 sulfaturia).ti,ab.	0

9	(familial osseous dystrophy or keratosulfaturia or osteochondrodystrophia deformans).ti,ab.	0

10	(Mucinos?s or mucinase or Mucinex or mucinol or mucinolytic).ti,ab.	93

11	(Sly adj3 (disease$ or syndrome$)).ti,ab.	7

12	((San Filippo or Sanfilippo$) adj3 (disease$ or syndrome$)).ti,ab.	64

13	or/1-12	3763

14	((h?ematopoietic stem cell$ or HSC or peripheral blood stem cell$) adj3 (transplant$ or therap$)).ti,ab.	11617

15	(HSCT or HSCTs).ti,ab.	10870

16	14 or 15	16837

**17	13 and 16	101**

**ClinicalTrials.gov**

[**https://www.clinicaltrials.gov/**](https://www.clinicaltrials.gov/)

**Date searched: 17.12.24**

**Records found: 57**

*Expert search interface:*

(mucopolysaccharidosis OR mucopolysaccharidoses OR muco-polysaccharidosis OR muco-polysaccharidoses OR MPS OR chondroosteodysplasia OR chondroosteodystrophy OR chondroosteoplasia OR dysostosis multiplex OR gargolylism OR gargoylism OR helmholtz harrington syndrome OR iduronidase deficiency syndrome OR lipochondrodystrophy OR lipochondrodystrophies OR mckusick 25280 OR Hurler OR Hurlers OR Hunter OR Hunters OR Morquio OR Maroteaux Lamy OR San Filippo OR Sanfilippo OR Mucinosis OR Mucinoses OR mucinase OR scheie OR hurlerscheie OR hurlersscheie OR pfaundlerhurler OR UllrichScheie) AND (hematopoietic stem cell transplant OR haematopoietic stem cell transplant OR hematopoietic stem cell transplants OR haematopoietic stem cell transplants OR haematopoietic stem cell therapy OR haematopoietic stem cell therapies OR hematopoietic stem cell therapy OR hematopoietic stem cell therapies OR HSCT OR HSCTs)

**World Health Organization International Clinical Trials Registry Platform (ICTRP)**

[**https://trialsearch.who.int/**](https://trialsearch.who.int/)

**Date searched: 17.12.24**

**Records found: 13**

(mucopolysaccharidosis OR mucopolysaccharidoses OR muco-polysaccharidosis OR muco-polysaccharidoses OR MPS OR chondroosteodysplasia OR chondroosteodystrophy OR chondroosteoplasia OR dysostosis multiplex OR gargolylism OR gargoylism OR helmholtz harrington syndrome OR iduronidase deficiency syndrome OR lipochondrodystrophy OR lipochondrodystrophies OR mckusick 25280 OR Hurler OR Hurlers OR Hunter OR Hunters OR Morquio OR Maroteaux Lamy OR San Filippo OR Sanfilippo OR Mucinosis OR Mucinoses OR mucinase OR scheie OR hurlerscheie OR hurlersscheie OR pfaundlerhurler OR UllrichScheie) AND (hematopoietic stem cell transplant OR haematopoietic stem cell transplant OR hematopoietic stem cell transplants OR haematopoietic stem cell transplants OR haematopoietic stem cell therapy OR haematopoietic stem cell therapies OR hematopoietic stem cell therapy OR hematopoietic stem cell therapies OR HSCT OR HSCTs)

**Orphanet**

[**https://www.orpha.net/**](https://www.orpha.net/)

**Date searched: 17.12.24**

**Records found: 30**

*Research Projects*

<https://www.orpha.net/en/research-trials/research-projects>

*- Searched for Mucopolysaccharidosis*

ORPHA:93473 Hurler syndrome – 2 results

ORPHA:93476 Hurler-Scheie syndrome – 0 results

ORPHA:579 Mucopolysaccharidosis type 1 – 13 results

ORPHA:93474 Scheie syndrome – 0 results

ORPHA:79269 Sanfilippo syndrome type A – 0 results

ORPHA:79270 Sanfilippo syndrome type B – 3 results

ORPHA:79271 Sanfilippo syndrome type C – 1 result

ORPHA:79272 Sanfilippo syndrome type D – 0 results

*Clinical Trials*

<https://www.orpha.net/en/research-trials/clinical-trials>

*- Searched for Mucopolysaccharidosis*

ORPHA:93473 Hurler syndrome – 1 result

ORPHA:93476 Hurler-Scheie syndrome – 0 results

ORPHA:579 Mucopolysaccharidosis type 1 – 0 results

ORPHA:93474 Scheie syndrome – 0 results

ORPHA:79269 Sanfilippo syndrome type A – 8 results

ORPHA:79270 Sanfilippo syndrome type B – 2 results

ORPHA:79271 Sanfilippo syndrome type C – 0 results

ORPHA:79272 Sanfilippo syndrome type D – 0 results
